# Protective effect of botulinum toxin A after cutaneous ischemia-reperfusion injury

**DOI:** 10.1038/srep09072

**Published:** 2015-03-13

**Authors:** Akihiko Uchiyama, Kazuya Yamada, Buddhini Perera, Sachiko Ogino, Yoko Yokoyama, Yuko Takeuchi, Osamu Ishikawa, Sei-ichiro Motegi

**Affiliations:** 1 Department of Dermatology, Gunma University Graduate School of Medicine, Japan

## Abstract

Botulinum toxin A (BTX-A) blocks the release of acetylcholine vesicles into the synaptic space, and has been clinically used for aesthetic indications, neuromuscular disorders and hyperhidrosis. Several studies have demonstrated that BTX-A enhanced the blood flow and improved ischemia in animal models. Our objective was to assess the effects of BTX-A on cutaneous ischemia-reperfusion (I/R) injuries, mimicking decubitus ulcers. The administration of BTX-A in I/R areas significantly inhibited the formation of decubitus-like ulcer in cutaneous I/R injury mouse model. The number of CD31^+^ vessels and αSMA^+^ pericytes or myofibroblasts in wounds were significantly increased in the I/R mice treated with BTX-A. The hypoxic area and the number of oxidative stress-associated DNA-damaged cells and apoptotic cells in the I/R sites were reduced by BTX-A administration. In an *in vitro* assay, BTX-A significantly prevented the oxidant-induced intracellular accumulation of reactive oxygen species (ROS) in vascular endothelial cells. Furthermore, the administration of BTX-A completely suppressed the ulcer formation in an intermittent short-time cutaneous I/R injury model. These results suggest that BTX-A might have protective effects against ulcer formation after cutaneous I/R injury by enhancing angiogenesis and inhibiting hypoxia-induced cellular damage. Exogenous application of BTX-A might have therapeutic potential for cutaneous I/R injuries.

Ischemia-reperfusion (I/R) injury is characterized by the reperfusion of blood to previously ischemic tissue, which induces excessive cellular injury^1^,^2^. Reperfusion of blood into a hypoxic tissue can induce a cascade of inflammation, including the infiltration of leukocytes and macrophages and the production of proinflammatory cytokines, resulting in the damages of vascular and lymphatic endothelium, edema, capillary narrowing and the apoptosis and necrosis of tissues^3^,^4^.

Reactive oxygen species (ROS), such as H_2_O_2_ and NO, also play essential roles in the tissues damage induced by reperfusion^4^,^5^,^6^,^7^,^8^,^9^. I/R injury is associated with vascular infarction or vasospasm in various organs, such as the brain, heart, liver, kidneys and skin. There is also increasing evidence that I/R injury is associated with the pathogenesis of pressure ulcers, also known as decubitus ulcers^10^,^11^,^12^. Raynaud's phenomenon (RP) is commonly observed in response to cold or emotional stress in patients with connective tissue diseases, especially systemic sclerosis. RP is an episodic vasospasm of peripheral blood vessels that results from the dysregulation of vasoconstriction and vasodilatation, suggesting that I/R injury might be involved in the pathogenesis of severe pain and paresthesia of the fingers, as well as the formation of digital ulcers^13^,^14^.

Botulinum toxin (BTX) is a polypeptide produced by the bacterium *Clostridium botulinum*, which contains a protease that plays an active role in inhibiting the acetylcholine release at the neuromuscular junction and the eccrine sweat glands. There are seven (A–G) serotypes of BTX. Type A has been extensively studied and clinically used. Recently, the therapeutic indications of BTX-A have been expanded for axial hyperhidrosis, blepharospasm, facial spasms, cervical dystonia, spasms of the extremities and also for aesthetic indications of facial wrinkles. In addition, there is a broad spectrum of other indications for migraine, achalasia, urinary bladder dysfunction and anal fissures^15^,^16^,^17^,^18^,^19^.

In *in vitro* assays, several reports demonstrated that BTX-A enhanced the blood flow and survival of ischemic skin flaps using animal cutaneous flap models^20^,^21^,^22^,^23^. With respect to I/R injury and BTX-A, Küçüker *et al.* showed that BTX-A application suppressed apoptosis and the tissue levels of malonyl dialdehyde (MDA) and nitric oxide end products (NOx) in a rat model of skeletal muscle I/R injury^24^. However, to the best of our knowledge, there have been no studies of the possible effects of BTX-A on cutaneous I/R injuries.

Several case reports have recently demonstrated the beneficial effect of BTX-A in patients with RP, including the enhancement of blood flow and improvement of the digital ulcers, pain and paresthesia of the fingers^25^,^26^,^27^,^28^,^29^,^30^. However, there has been no experimental evidence of the beneficial effects of BTX-A on the I/R injury associated with RP-induced ulcers using animal models. In this study, we examined the effects of BTX-A on cutaneous I/R injuries in two experimental conditions that mimic decubitus ulcers and RP-induced cutaneous ulcers, respectively, and aimed to clarify the mechanisms underlying the protective effect of BTX-A against cutaneous I/R injury.

## Results

### Botulinum toxin A protected against ulcer formation in a decubitus ulcer-like I/R injury mouse model

**Figure 1:**
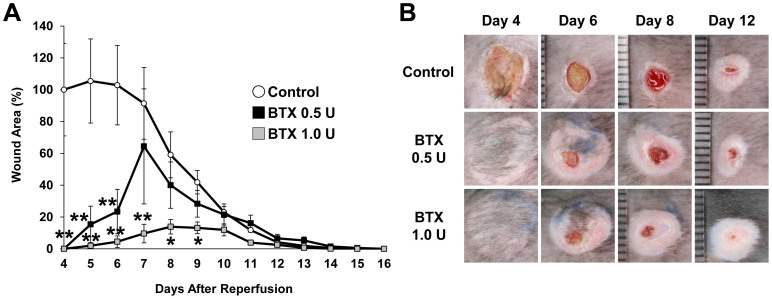
Botulinum toxin A protected ulcer formation in decubitus ulcer-like I/R injury mice model. (A) Percent wound area at each time point relative to the wound area in control mice at 4 days after reperfusion (n = 7 for each time point and groups). All values represent mean ± SEM. **P < 0.01, *P < 0.05. (B) Photographs of wound after cutaneous I/R in control or BTX treated mice at 4, 6, 8, and 12 days after reperfusion.

First, to assess the effects of BTX-A on the development of cutaneous pressure ulcers after I/R injury *in vivo*, we compared the wound area in a decubitus ulcer-like I/R injury mouse model treated with or without BTX-A. BTX-A was injected into the dermis at the I/R site 24 hours before the beginning of I/R. The administration of BTX-A significantly inhibited the formation of cutaneous pressure ulcers after I/R injury (Figures 1A, B). At four days after reperfusion, cutaneous ulcers had developed due to I/R injury in the control mice. However, the administration of BTX-A completely protected the mice from ulcer formation in the I/R area (Figures 1A, B). Furthermore, from four to nine days after reperfusion, the wound areas in 1.0 U BTX-A-treated mice were significantly smaller than those in control mice. These results suggest that BTX-A might have the potential to prevent the development of cutaneous pressure ulcers after I/R injury.

### Botulinum toxin A protected against the reduction of vascularity induced by cutaneous I/R injury

**Figure 2:**
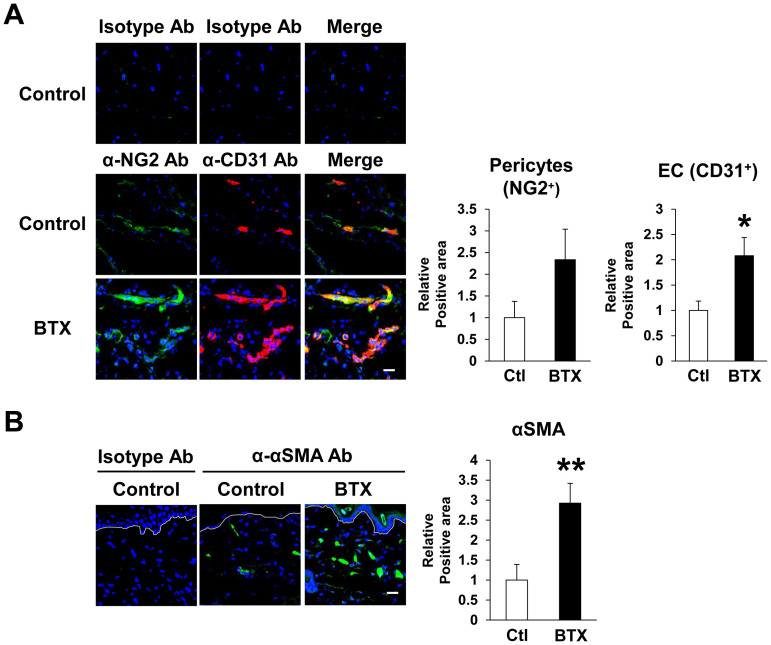
Botulinum toxin A protected the reduction of vascularity by cutaneous I/R injury. (A) The amount of CD31^+^ EC and NG2^+^ pericytes in cutaneous I/R area at 4 days after reperfusion. (B) The amount of αSMA^+^ myofibroblast or pericytes in cutaneous I/R area at 4 days after reperfusion. Quantification of the CD31^+^, NG2^+^ and αSMA^+^ areas in 6 random microscopic fields from the periphery of I/R area in n = 3 mice per groups was performed using Image J software. Positive area in control mice was assigned a value of 1. Values represent mean ± SEM. **P < 0.01, *P < 0.05. Scale bar = 20 μm.

Kasuya *et al.* recently reported that cutaneous I/R injury induced the suppression of the luminal areas of blood vessels and lymphatic vessels, as well as inducing hypoxia and oxidative stress in the I/R site^4^. In addition, several studies using animal cutaneous flap models demonstrated that BTX-A prevented the collapse of the peripheral vessels in the cutaneous flap and increased the blood flow and survival of the flap^20^,^21^,^22^,^23^. Based on these previous studies, we investigated the effects of BTX-A on the vascularity in the I/R area in a cutaneous I/R injury model. At four days after reperfusion, the numbers of CD31^+^ endothelial cells in I/R areas in BTX-A-treated mice were significantly increased compared to those in control mice (Figure 2A). The numbers of NG2^+^ pericytes tended to be higher than those in control mice. The numbers of αSMA^+^ pericytes or myofibroblasts in I/R areas in BTX-A-treated mice were significantly increased compared to those in control mice (Figure 2B). We confirmed that the stainings using isotype control antibodies were negative. We additionally examined the effect of BTX-A on the vascularity in the I/R area at earlier time point after I/R, such as at 1 hour after reperfusion. The amount of CD31^+^ endothelial cells, NG2^+^ pericytes and αSMA^+^ pericytes or myofibroblasts in I/R areas in BTX-A-treated mice at 1 hour after reperfusion were significantly increased compared to those in control mice (Supplemental Figure S1A,B). These results suggest that BTX-A might have a preventive effect against the reduction of vascularity by cutaneous I/R injury.

### Botulinum toxin A reduced the hypoxic area after cutaneous I/R injury

**Figure 3:**
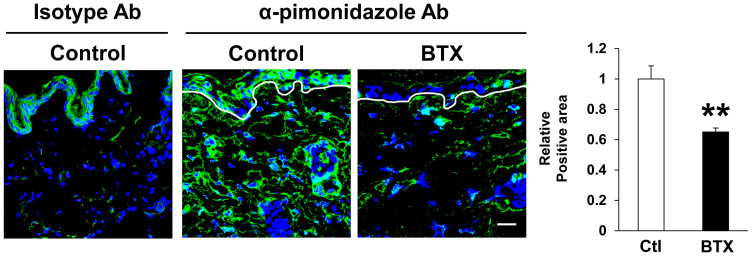
Botulinum toxin A reduced hypoxic area after cutaneous I/R injury. The amount of pimonidazole^+^ hypoxic area in cutaneous I/R site at 1 day after reperfusion. Quantification of the pimonidazole^+^ areas in 8 random microscopic fields from the center of I/R area in n = 3 mice per groups was performed using Image J software. Positive area in control mice was assigned a value of 1. Values represent mean ± SEM. **P < 0.01. Scale bar = 20 μm.

To examine the influence of BTX-A on the tissue hypoxic area in the cutaneous I/R area after I/R injury in mice, immunofluorescent staining of the hypoxic area using an antibody against pimonidazole, a marker of hypoxia, was performed with skin tissue sections from the I/R area. At one day after reperfusion, the hypoxic area at the I/R site in BTX-A-treated mice was significantly reduced compared to that in control mice (Figure 3). We confirmed that the staining using isotype control antibody was negative. We additionally examined the effect of BTX-A on the hypoxic area in the I/R area at earlier time point after I/R, such as at 1 hour after reperfusion. The amount of hypoxic area in I/R areas in BTX-A-treated mice at 1 hour after reperfusion was significantly reduced compared to that in control mice (Supplemental Figure S1C). These results suggest that BTX-A might have a preventive effect against hypoxia by cutaneous I/R injury in the I/R area.

### Botulinum toxin A protected against DNA damage after cutaneous I/R injury

**Figure 4:**
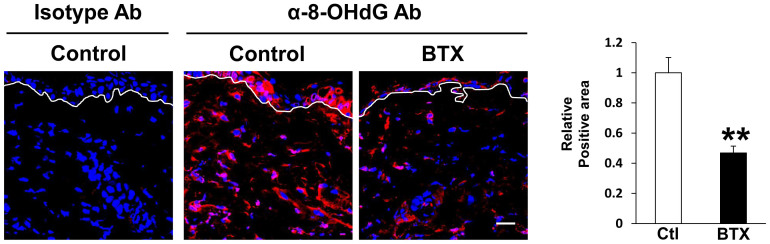
Botulinum toxin A protected DNA damages after cutaneous I/R injury. The amount of 8-OHdG^+^ DNA damaged area in cutaneous I/R site at 1 day after reperfusion. Quantification of the 8-OHdG^+^ areas in 6 random microscopic fields from the center of I/R area in n = 3 mice per groups was performed using Image J software. Positive area in control mice was assigned a value of 1. Values represent mean ± SEM. **P < 0.01. Scale bar = 20 μm.

Kasuya *et al.* demonstrated that ROS were produced by I/R injury and created 8-hydroxy-2- deoxyguanosine (8-OHdG), a useful marker of oxidative stress-associated DNA damage, in the DNA of tissue-resident cells^4^. Therefore, we examined the effects of BTX-A on the DNA damage after cutaneous I/R injury by performing immunofluorescent staining of 8-OHdG. At one day after reperfusion, the area with positive staining in the I/R area in BTX-A-treated mice was significantly reduced compared to that in control mice (Figure 4). We confirmed that the staining using isotype control antibody was negative. These results suggest that BTX-A might reduce oxidative stress due to cutaneous I/R injury.

### Botulinum toxin A reduced cell apoptosis after cutaneous I/R injury

**Figure 5:**
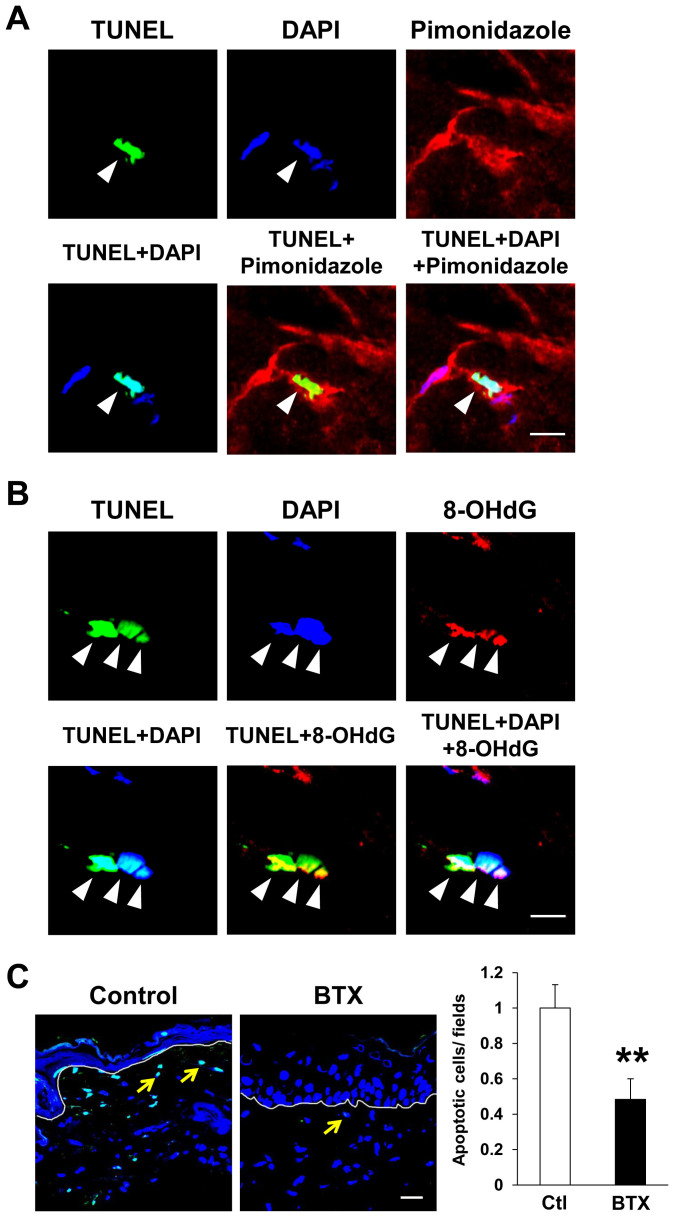
Botulinum toxin A reduced apoptotic cells after cutaneous I/R injury. (A) Stainings of TUNEL, DAPI and pimonidazole in cutaneous I/R area at 1 day after reperfusion. Stainings of TUNEL and DAPI double-positive nuclei were localized in pimonidazole^+^ hypoxic area (Arrowhead). Scale bar = 5 μm. (B) Stainings of TUNEL, DAPI and 8-OHdG in cutaneous I/R area at 1 day after reperfusion. Stainings of TUNEL were co-localized with both DAPI and 8-OHdG (Arrowhead). Scale bar = 5 μm. (C) The number of apoptotic cells in I/R site at 1 day after reperfusion was determined by counting both TUNEL and DAPI positive cells (Arrow). Values were determined in 6 random microscopic fields from the center of I/R area in n = 3 mice per groups. The number of apoptotic cells in control mice was assigned a value of 1. Values represent mean ± SEM. **P < 0.01. Scale bar = 20 μm.

ROS induced by I/R injury causes apoptosis and subsequent secondary necrosis, and these responses induce inflammation in the I/R area^31^,^32^. At first, we examined whether hypoxia and/or oxidative stress are related to the apoptosis in I/R area at 1 day after reperfusion. The stainings of TUNEL and DAPI double-positive nuclei were localized in pimonidazole^+^ hypoxic area (Figure 5A). In addition, the stainings of TUNEL were co-localized with both DAPI and 8-OHdG (Figure 5B). These results suggest that hypoxia and oxidative stress by I/R injury might be associated with apoptosis in cutaneous I/R area. We next examined the influence of BTX-A on the number of apoptotic cells in the I/R areas in mice. One day after reperfusion, the number of apoptotic cells in the I/R areas in BTX-A-treated mice were significantly lower than those in control mice (Figure 5C). These results suggest that BTX-A might reduce the apoptosis induced by cutaneous I/R injury.

### Botulinum toxin A reduced the oxidant-induced intracellular accumulation of ROS in vascular endothelial cells

**Figure 6:**
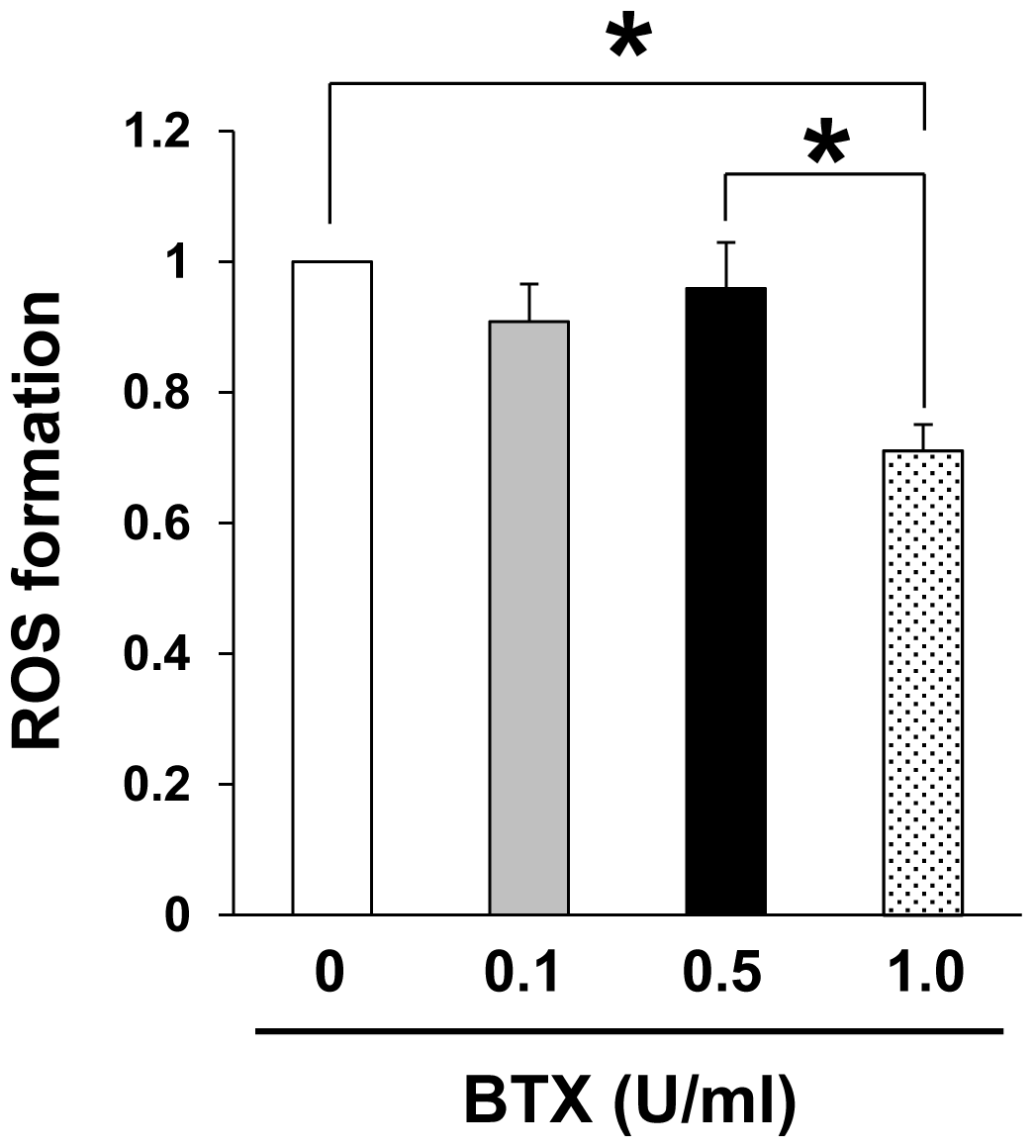
Botulinum toxin A reduced oxidant-induced intracellular accumulation of ROS in vascular endothelial cells. Quantification of H_2_O_2_-induced intracellular ROS production in vascular endothelial cells treated with or without BTX. ROS formation in cells without BTX treatment was assigned a value of 1. Values represent mean ± SEM. n = 4 in each group. *P < 0.05.

To examine the effects of BTX-A on the oxidative stress affecting vascular endothelial cells, we next examined the effects of BTX-A on H_2_O_2_-induced intracellular ROS accumulation in vascular endothelial cells using human umbilical vein endothelial cells (HUVECs) *in vitro*. BTX-A suppressed the H_2_O_2_-induced intracellular ROS accumulation in the HUVECs in a concentration-dependent manner (Figure 6), suggesting that BTX-A might reduce oxidative stress in endothelial cells after I/R injury.

### Botulinum toxin A protected against ulcer formation in an intermittent short-time cutaneous I/R injury

**Figure 7:**
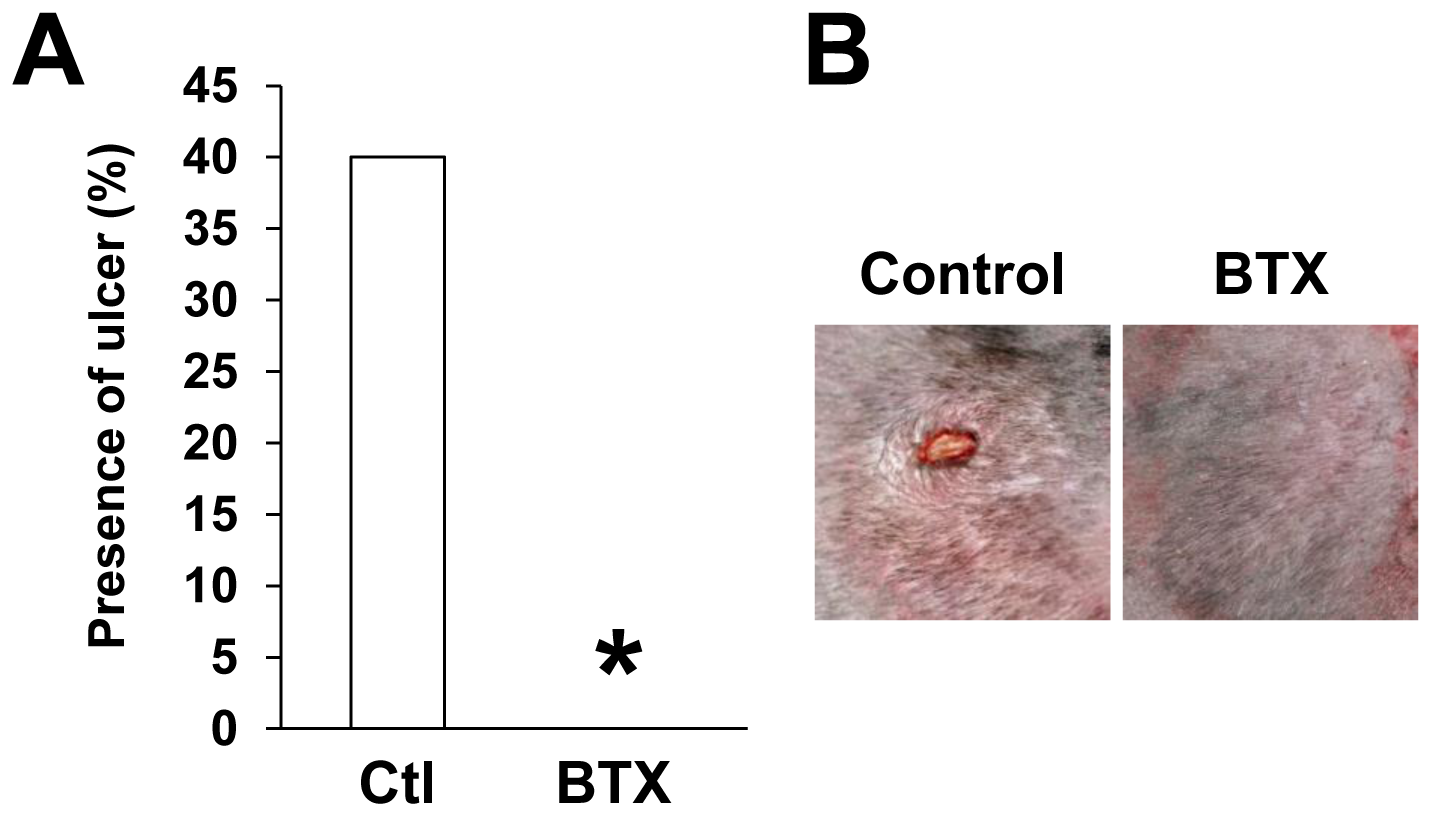
Botulinum toxin A protected ulcer formation in an intermittent short-time cutaneous I/R injury. (A) The frequency of the presence of skin ulcers in I/R area at 4 days after 3 cycles of I/R injury (n = 10 in each group). *P < 0.05. (B) Photographs of mice back skin after cutaneous I/R in control or BTX treated mice at 4 days after reperfusion.

We introduced a new cutaneous I/R injury model, which is consisted of three I/R cycles; three-hour period of magnet placement, followed by a reperfusion period of 3 hours, suggesting that this intermittent short-time cutaneous I/R injury may mimic the pathogenesis of RP. Forty percent of the mice developed small skin ulcers after these short I/R cycles by four days after the I/R cycles (Figure 7). The administration of BTX-A completely protected against the ulcer formation after I/R injury (Figure 7). These results confirmed that BTX-A might also prevent the development of cutaneous ulcers induced by the intermittent short-time cutaneous I/R injury.

## Discussion

I/R injury induces inflammation, including the infiltration of leukocytes and macrophages and the production of proinflammatory cytokines, and leads to the dysfunction of vascular endothelium, capillary narrowing, vascular infarction and vascular spasm. These vascular damages cause hypoxia and oxidative stress in tissues, resulting in the apoptosis and necrosis of these tissues^1^,^2^,^3^,^4^. In the present study, we first assessed the vascular damage induced by I/R injury, and found that the numbers of CD31^+^ endothelial cells and αSMA^+^ pericytes or myofibroblasts in I/R areas in BTX-A-treated mice were significantly higher than those in control mice. These results suggest that BTX-A could protect against vascular damages by I/R injury.

The mechanism(s) by which BTX-A protects against the vascular damage induced by I/R injury is unclear. It has been recognized that BTX-A has vasodilatory effects through sympathetic inhibition at the neuromuscular junction. Several previous studies have reported that treatment of the microvasculature with BTX-A causes an increase in the arteriolar diameter and a subsequent increase in blood flow^20^,^21^,^22^,^23^. In addition, pre-treatment with BTX-A was associated with a lower rate of arterial and venous thrombosis in an animal model microanastomosis^31^. The damage of the vascular endothelium, including capillary narrowing, vascular infarction and spasm, is associated with the pathogenesis of I/R injury. In addition, endothelium-dependent relaxation is decreased by the damage to the vascular endothelium by I/R injury. These findings suggest that the vasodilation and the inhibition of thrombosis and vasospasm by BTX-A might be involved in the protective effects of BTX-A against the vascular damage caused by I/R injury.

It has been reported that hypoxic insult to vascular endothelial cells by I/R injury resulted in leukocyte-endothelial cell adhesion and neutrophil migration through the endothelial barrier^32^,^33^. Reactive oxygen species (ROS), such as H_2_O_2_ and NO, also play essential roles in the tissue damage^4^,^5^,^6^,^7^,^8^,^9^. We herein demonstrated that BTX-A reduced oxidative stress in vascular endothelial cells *in vitro*. These results suggest that BTX-A might protect against the hypoxic insult to the vascular endothelial cells, and these effects mediated by BTX-A might provide us with new insight into the mechanisms by which BTX-A protects against I/R injury. However, further investigations are required to elucidate the precise mechanisms by which BTX-A can reduce oxidative stress.

This is the first investigation to examine the effects of BTX-A against cutaneous I/R injury. Using two experimental conditions and an *in vitro* study, we identified the mechanisms underlying the protective effects of BTX-A against cutaneous I/R injury; (i) protection against the reduction of vascularity by I/R injury, (ii) reduction of the hypoxic area, oxidative stress and apoptosis of cells *in vivo* and (iii) reduction of oxidative stress-induced ROS in vascular endothelial cells *in vitro*. These results suggest that BTX-A might have the potential to prevent the ulcer formation induced by cutaneous I/R injury.

Recently, several studies have reported that the administration of BTX-A significantly improved the symptoms of RP, such as the pain, paresthesia of fingers and digital ulcers^25^,^26^,^27^,^28^,^29^,^30^. One of the mechanisms was reported to be its established acetylcholine-mediated vascular smooth muscle paralysis, which results in the inhibition of spasm and vascular contraction. Another mechanism is thought to be due to the fact that BTX-A inhibits the expression of adrenergic receptors in the vessel walls and blocks the release of norepinephrine and various other neuropeptides, such as calcitonin gene-related peptide (CGRP), glutamate and substance P, which are increased in chronic nerve irritation and pain, and can exacerbate these symptoms^28^,^29^,^34^,^35^. There had previously been no experimental evidence of the beneficial effects of BTX-A against I/R injury associated with RP-induced ulcers using animal models. In this study, we introduced a new experimental condition; an intermittent short-time I/R cutaneous injury. This condition may mimic RP-induced cutaneous ulcers. We demonstrated that the administration of BTX-A completely protected against the ulcer formation after I/R injury. These results confirmed that BTX-A might have preventive and/or therapeutic potential with regard to the development of cutaneous ulcers due to the intermittent I/R as seen in RP.

Taken together, the present findings indicate that BTX-A suppresses the formation of skin ulcers induced by cutaneous I/R injury by protecting against vascular damage, suppressing hypoxia, decreasing oxidative stress and preventing apoptosis. In addition, BTX-A could be expected to be effective for at least three to six months in humans^25^,^26^,^27^,^28^,^29^,^30^, therefore, exogenous BTX-A administration has possible long-term preventive and therapeutic potential for patients with cutaneous I/R injuries, including decubitus ulcers and RP-induced digital ulcers.

## Methods

### Animals

All experiments were approved by the Ethical Committee for Animal Experiments of the Gunma University Graduate School of Medicine, and carried out in accordance with the approved guidelines. C57BL/6 mice were purchased from the SLC (Shizuoka, Japan). Eight- to 12-week-old female mice were used for all experiments. Mice were maintained in the Institute of Experimental Animal Research of Gunma University under specific pathogen-free conditions. Mice were handled in accordance with the animal care guidelines of Gunma University.

### Antibodies

Antibodies (Abs) and their sources were as follows: rat anti-mouse CD31 monoclonal Ab (mAb) (MEC13.3; BD Bioscience, San Jose, CA), rabbit anti-mouse NG2 polyclonal Ab (pAb) (Millipore, Billerica, MA), FITC-conjugated mouse anti-αSMA mAb (Sigma, St Louis, MO), goat anti-8-OHdG pAb (abcam, Cambridge, UK). Alexa 488-, Alexa 568-conjugated secondary Abs were obtained from Invitrogen (Carlsbad, CA).

### I/R cycles

We used 2 types of cutaneous I/R cycle mice models. The cutaneous I/R model was performed according to previously published protocols^36^,^37^,^38^. Briefly, mice were anesthetized, and hair was shaved and cleaned with 70% ethanol. The dorsal skin was gently pulled up and trapped between two round ferrite magnetic plates that had a 12-mm diameter (113 mm^2^) and 5 mm thick, with an average weight of 2.69 g and 1180 G magnetic forces (NeoMag Co, Ichikawa, Japan). Epidermis, dermis, subcutaneous fat layer and subcutaneous loose connective tissue layer, but not muscles, were pinched by magnetic plates. This process creates a compressive pressure of 50 mmHg between the two magnets^36^,^37^. In the analysis of “decubitus ulcer-like cutaneous I/R model”, dorsal skin was trapped between magnetic palates for 12 hours, and then plates were removed. Mice were not immobilized, and not anesthetized during ischemia. All of the mice developed two round ulcers separated by a bridge of normal skin. In the analysis of “intermittent short-time cutaneous I/R model”, 3 I/R cycles were performed in each mouse. A single IR cycle consisted of a 3-hour period of magnet placement, followed by a release or rest period of 3 hours. To assess the effects of BTX-A (BOTOX VISTA®, Allergan Pharmaceuticals, Irvine, CA) on the development of ulcers after cutaneous I/R injury, 0.5 U or 1.0 U BTX-A per 200 μl 0.9% saline or 200 μl of saline as a control were injected into the dermis in the I/R site 24 hours before the beginning of I/R cycles. For analysis, each wound sites were digitally photographed at the indicated time points after wounding, and wound areas were measured on photographs using Image J (version1.48, NIH, Bethesda, MD) as previously reported^39^.

### Histological examination and Immunofluorescence staining

Immunofluorescence staining of frozen sections and analyses were performed according to previously described protocols^39^,^40^. Murine skins were removed and 4 μm frozen sections were prepared and fixed in 4% PFA in PBS for 30 minutes. After blocking with 3% dry milk-PBS supplemented with 5% normal donkey serum or 5% normal goat serum for 1 hour at room temperature, sections were stained with Abs of interest followed by Alexa 488-, Alexa 568-conjugated secondary Abs. Sections were counterstained with 4,6-diamidino-2-phenylindole (DAPI) to visualize nuclei, mounted in ProLong Gold antifade reagent (Invitrogen).

### Assessment of tissue hypoxia

Hypoxic areas after cutaneous I/R injury in I/R site were detected using the Hypoxyprobe™-1 Omni kit (Hypoxyprobe, Inc., Burlington, MA) according to the manufacture's protocol. Pimonidazole HCl was injected intraperitoneally (60 mg/kg) 30 minutes before the sacrifice of the mice. Murine skins were removed and 4 μm frozen sections were prepared and fixed cold acetone (4°C) for 10 minutes. Sections were incubated overnight at 4°C with rabbit anti-pimonidazole Ab (PAb2627) diluted 1:20 in PBS containing 0.1% bovine serum albumin and 0.1% Tween 20. Sections were incubated for 1 hour with Alexa 488-conjugated secondary Ab. Images (8 fields/section) were taken and visualized with a FV10i-DOC confocal laserscanning microscope (Olympus). The positive area was determined by Image J (version1.48, NIH, Bethesda, MD) in the field (x600) as previously reported^39^,^40^.

### ROS detection assay *in vitro*

HUVEC were purchased from ATCC (Manassan, VA). HUVEC were maintained in EBM-2 basal medium (Lonza, Basel, Switzerland) supplemented with EGM-2 Single Quot Kit Suppl. & Growth Factors (Lonza). HUVECs (2.5 × 10^4^ cells) were cultured in OptiPlate™-96F microplate (Perkin Elmer, Waltham, MA). Cells were incubated in the medium with or without BTX-A (0, 0.1, 0.5, 1.0 U/ml FBS(-) DMEM) at 37°C for 24 hours. Cells were stimulated with 0.25 mM H_2_O_2_ (100 μl/well) for 2 hours, and then ROS levels were measured with DCFDA Cellular ROS Detection Assay Kit (abcam) according to the manufacturer's protocol. Fluorescence was detected by plate reader (Perkin Elmer).

### Apoptosis assay

The presence of apoptotic cells in the skin sections were assessed 4 days after reperfusion using terminal deoxynucleotide transferase dUTP nick end-labeling (TUNEL) staining kit (Roche Diagnostics, Indianapolis, IN) according to the manufacturer's protocols. Images (6 fields/section) were taken and visualized with a FV10i-DOC confocal laserscanning microscope (Olympus). The number of apoptotic cells was determined by counting TUNEL and DAPI double positive nuclei in the field (x600) as previously reported^39^,^40^.

### Statistical analysis

*P* values were calculated using the Student's *t*-test (two-sided), Chi-square test analysis or by analysis of one-way ANOVA followed by Bonferroni's post test as appropriate. Error bars represent standard errors of the mean, and numbers of experiments (n) are as indicated.

## Supplementary Material

Supplementary Information Supplemental Figure S1
