# Supplementary material for: Protective effect of botulinum toxin A after cutaneous ischemia-reperfusion injury
Source: Sci Rep. 2015 Mar 13;5:9072. doi: 10.1038/srep09072 (PMC5390917; doi:10.1038/srep09072)
Supplement: Supplementary Information — Supplemental Figure S1 [file srep09072-s1.doc]

**Supplementary Data**

**Protective effect of botulinum toxin A after cutaneous ischemia-reperfusion injury**

Akihiko Uchiyama, Kazuya Yamada, Buddhini Perera, Sachiko Ogino, Yoko Yokoyama, Yuko Takeuchi, Osamu Ishikawa & Sei-ichiro Motegi*

Department of Dermatology, Gunma University Graduate School of Medicine, Japan

**Figure S1. Botulinum toxin A protected the reduction of vascularity and reduced hypoxic area at earlier time point after I/R injury.** (A) The amount of CD31^+^ EC and NG2^+^ pericytes in cutaneous I/R area at 1 hour after reperfusion. (B) The amount of αSMA^+^ myofibroblast or pericytes in cutaneous I/R area at 1 hour after reperfusion. Quantification of the CD31^+^, NG2^+^ and αSMA^+^ areas in 6 random microscopic fields in n=3 mice per groups was performed using Image J software. Positive area in control mice was assigned a value of 1. Values represent mean ± SEM. ***P*<0.01. Scale bar = 20 μm. (C) The amount of pimonidazole^+^ hypoxic area in cutaneous I/R site at 1 hour after reperfusion. Quantification of the pimonidazole^+^ areas in 8 random microscopic fields in n=3 mice per groups was performed using Image J software. Positive area in control mice was assigned a value of 1. Values represent mean ± SEM. ***P*<0.01. Scale bar = 20 μm.
